# Memorial Celebration of the Fiftieth Anniversary of the Discovery of Anæsthesia

**Published:** 1895-01

**Authors:** 


### MEMORIAL CELEBRATION OF THE FIFTIETH ANNIVERSARY OF THE DISCOVERY OF ANÆSTHESIA.

The celebration of the discovery of anæsthesia, in Philadelphia, December 11, 1894, will take rank as being one of the most important, if not the greatest celebration that dentistry has ever attempted in this country, the Columbian Dental Congress excepted.

When the American Dental Association, at Old Point Comfort in August last, decided to hold this in honor of Wells, the discoverer of anæsthesia, it was with some doubt as to the possibility of interesting the profession generally throughout the United States, sufficiently so, at least, to induce them to leave their homes at a busy season of the year. The result shows that this was, to a large degree, an error, for the representation from fourteen States was not only good as to number, but also represented in quality the best thought in dentistry.

The meeting assembled promptly at 2 P.M. on Tuesday, December 11, and notwithstanding the inclemency of the weather there was a large and enthusiastic audience present.

The meeting was opened by Dr. J. Y. Crawford, President of the American Dental Association, in a few brief remarks explaining the reasons that influenced the American Dental Association to call this memorial meeting, and he then introduced Dr. Thomas Fillebrown, of Boston, who presented the history of the development of anæsthesia from the earlier periods of human history until it culminated in the final discovery by Wells. It was a masterly argument, and held the undivided interest of the audience until its close, and will bear careful perusal and thoughtful consideration by our readers.

(The address will be found on page 1.)

The President then presented Dr. James E. Garretson, of Philadelphia, Professor of Anatomy and Surgery, Philadelphia Dental College. Dr. Garretson took the philosophical and humanitarian side of the question. His scholarly presentation of the deeper thoughts underlying this discovery was given very effectively. The contrast drawn between the surgery of to-day, illustrated by a recent case of his own and that of the mother and the child, was an impressive piece of work, and coming from the great surgeon as an experience, was worthy of being embalmed in the memories of those present.

At the conclusion of this address Dr. G. Q. Colton, of New York, was discovered in the audience, and was at once invited by the President to a seat upon the platform as the surviving representative of that now historical experience in which Wells had his own tooth extracted, he, Colton, giving the nitrous oxide. In a remarkably clear voice for a man of eighty-one he detailed the facts as he knew them, greatly to the interest of those present. It seemed almost impossible that a man could be present and take part in this celebration of an event, in which he was one of the principal actors, fifty years ago. It seemed to bridge, most satisfactorily, the present with the stirring streams of life moving during the first half of the present century towards a higher development of dental work.

Mr. Charles Wells, son of the discoverer, was introduced, and expressed his thanks for the honor, but excused himself from speaking at that meeting.

Dr. L. D. Shepard, of Boston, then moved that the meeting adopt a reaffirmation of its appreciation of Dr. Horace Wells's discovery. This was approved.

Dr. Robert Huey, of Philadelphia, moved that a committee be appointed to consider the erection of a monument to Dr. Wells in Washington, D. C. This was also adopted, and Dr. J. D. Thomas, of Philadelphia, was appointed treasurer.

The meeting then adjourned to meet at the banquet, Union League Club House of Philadelphia, at 6.30 in the evening.

This now historic building never seemed so inviting as it did on that evening as the guests assembled in its spacious library room. The professions were well represented by one or more from each. The brilliant lights and profusion of flowers combined to give the occasion its appropriate setting. The interchange of greetings between old professional friends and the introductions to new ones made the waiting hour a very agreeable part of the day's work.

The opening of the large doors and the march into the magnificent dining-hall of the League was a surprise to all, and loud expressions of the beauty of the scene were heard on every side. The hall is capable of accommodating large numbers. Its perfect appointments made a most effective background to the beautifully decorated tables. The walls were banked with palms, flowers everywhere, giving fragrance, beauty, and poetry to the scene. The dazzling electric lights reflected on "crystal touching crystal" of the costly chandelier seemed to send a glow of pleasure to every guest, and doubtless also a feeling of satisfaction to the committee having the celebration in charge that their labors were receiving a due reward. The expressions of regret were general that the scene could not have been witnessed by every thoughtful dentist of the land. It was very impressive, and the speeches of the evening seemed to reflect the consciousness of an overpowering influence for good. It was a glorification not alone of Dr. Wells, but it represented that nobler humanitarian influence that seems to be developed on special occasions as an evidence that underlying all selfishness there is an under-strata of eternal good that somewhere and somehow is destined to regenerate the race.

The menu was rapidly passed over. It was excellent in quality and well served. At the conclusion of the gastronomic part of the proceedings, Professor E. T. Darby called the attention of those present to the importance of the occasion and its possible far-reaching effect. Horace Wells was a citizen of Hartford, and it was fitting that a distinguished representative from the State of Connecticut, United States Senator General Joseph B. Hawley, should respond to the toast "The Horace Wells Discovery."

The Senator took up the history of the introduction of anæsthesia, and said he had never before been so impressed with the importance of this great subject as upon the present occasion. He pictured the sufferings of the people of past centuries, and contrasted these with the present freedom from pain. The feelings of the residents of the city of Hartford were graphically described, the enthusiasm of the old men, prominent in that city's life, who came out the evening previous at Hartford's celebration of the discovery of anæsthesia, and remained until after one o'clock to assist in placing a memorial tablet to the honor of her great citizen Horace Wells. He mentioned the work of Hon. Truman Smith, and closed by expressing the deep pleasure it gave him to take part in this celebration.

Professor Darby then called on Dr. James Truman, Professor of Dental Pathology, etc., University of Pennsylvania, of Philadelphia, to respond to the toast "Anæsthesia as a Dental Discovery."

He began his remarks by alluding to the story of Hans Christian Andersen of "The Ugly Duckling," and compared this experience with that of dentistry. It was born late into the professions. It had undergone contumely and criticism, but nevertheless it had grown and developed,—if not into the beautiful and graceful swan, had, at least, advanced into the higher walks of professional life. This, he conceived, was demonstrated in innumerable ways, but in no one more striking than the fact that fourteen States were represented at this banquet. When this was remembered as meaning personal sacrifice to all and days of travel to many, it proved to his mind that the dental profession was beginning to learn the great lesson of what is meant by professional spirit, and this evidence rejoiced his heart on this occasion.

He elaborated on the work of Horace Wells. His unselfishness; his desire to spread this blessing broadcast into "the greater world," as Goethe loved to call it.

Horace Wells was the discoverer of anæsthesia. When Dr. B. W. Richardson, of England, in a recent article in *Longman's Magazine*, attempted to tear the mantle from Wells's shoulders and place it on Sir Humphry Davy, he made the blunder of his great life.

Dr. Truman then quoted from Richardson's account of the condition of surgery just prior to the introduction of anæsthesia. The anxiety of the surgeon, the horror of the students, and the terror of the patients were given by Dr. Richardson with a vividness worthy a master. His graphic account of the reception of the first news received from Massachusetts Hospital, and of the first operation performed in his presence in England; and yet, at this time, England had forgotten that Davy had ever mentioned nitrous oxide in connection with the amelioration of pain.

Dr. Truman compared this work to other discoveries, and spoke of the inspirations floating in the mental ether of the world and falling like the dews of heaven, as in the parable, some on rocky soil, some on poor, and others on fruitful soil. It fell to Priestley, and there was no germination; it fell on Davy, and ended in a weakly plant,—a dream; it fell upon Horace Wells, and the plant grew, to the comfort and happiness of the nations.

Dentistry, in his view, had not reaped much benefit from this discovery. When Horace Wells lived and worked this profession was in a transition state. Anæsthesia was important then for both patient and operator, but dentistry has now far outgrown this condition, and beyond the necessity of extracting teeth to the extent then required by existing knowledge.

He said we might well adopt the old Delphian idea and hang the "leaden forceps" in our temples—our offices,—an emblem to us that teeth are not to be removed until such an instrument could extract them.

He then pictured the horrors of the battle-fields of past centuries, the terrors of the hospital, and the sufferings in the accidents of life, and said, "Who can translate these into words suitable for modern comprehension?

"The wail of the ages was voiced in the garden of Gethsemane when the Great Master of Human Thought, in anticipation of the cruel agonies of the cross, cried aloud, 'Father, let this cup pass from me.' Eighteen centuries passed into oblivion and there came no response, but near high noon on the nineteenth, in the New World of Columbus, in a humble home at Hartford, a 'still, small voice' was heard sounding above the wilderness of suffering; it was wafted over the deep waters; it echoed and re-echoed in joyous acclaim throughout the world, 'Lo, the cry of agony from the surgeon's knife is silenced forever!'

"We cannot deify thee, but we give thee most hearty thanks, and in our heart of hearts we enshrine thee, O Anæsthesia, goddess of our modern civilization, though not the first-born, the loveliest of all the children of discovery, in this our nineteenth century."

Dr. J. William White, Professor of Clinical Surgery, University of Pennsylvania, then responded to the sentiment "Anæsthesia as a Factor in the Evolution of Surgery." He paid a glowing tribute to Horace Wells, and said, "If this discovery did nothing more than blot out the suffering of the patient, it had justified a hundred times over this commemorative gathering. The brilliant thought of Wells," he remarked, "so broadened the field of surgery that it made possible the discoveries of Pasteur and other great men twenty-five years later."

Dr. H. C. Wood, Professor of Materia Medica, Pharmacy, and Therapeutics, of the University of Pennsylvania, spoke on "The Debt of Medicine to Anæsthesia." With his usual eloquence and energy he advocated the necessity for vivisection, but, he said, few would have the cruelty to injure animals without anæsthesia. In this sense has anæsthesia made progress possible with all the great bodies connected with medical science.

Over against medicine stands the ignorant public in opposition to this necessary method to advance medical knowledge.

It is a truth that medical men are not rewarded. No statues are erected to doctors; dollars and cents rule the world.

He said it was a great thing for Hartford to place a tablet in commemoration of the discovery of anæsthesia by Horace Wells, but why did not the city raise a monument to his memory and great services?

Senator Hawley explained that the city of Hartford had erected years ago a monument in the form of a full-length statue.

Colonel Alexander McClure, editor-in-chief of the Philadelphia *Times*, spoke on "The Mastery of Pain from the Stand-point of the Layman." He was greeted with much applause, and delivered one of his delightful after-dinner speeches, abounding in genial wit and appropriate allusions.

He took the position that anæsthesia gave to medicine the opportunity of attaining the highest art in surgery. Operations were performed to-day with ease and certainty impossible before its advent.

He deprecated the fact that the prejudices of the ignorant had, in a measure, dwarfed medical science under the belief that vivisection was cruel, but that in the end these would be conquered and the world would progress in spite of such opposing forces.

Dr. Wilbur F. Litch, Professor of Prosthetic Dentistry, Materia Medica, and Therapeutics, Pennsylvania College of Dental Surgery, followed on "The Development of our Knowledge of Anæsthesia."

He remarked that the occasion brought to his mind with renewed force the observation of Lecky, the distinguished English historian, that "It is probable that the American inventor of the first anæsthetic has done more for the real happiness of mankind than all the moral philosophers from Socrates to Mill."

He quoted the great French surgeon Velpeau as saying, in a work published in 1832, "To avoid pain under incisions is a chimera which is no longer pursued by any one. A cutting instrument and pain in operative surgery are two words which never present themselves separately to the mind of the patient, and of which he must of necessity admit the inevitable association," and commented upon Velpeau's changed attitude when in 1847, before the French Academy of Sciences, he gave an account of his successes with ether, and reported that he had, among other operations, "removed an enormous cancer from the thigh of a man who had not the slightest perception of the operation."

Dr. Litch affirmed that to Horace Wells more than to any other man the world was indebted for the beneficent discovery which made Velpeau's radical change of opinion and practice possible.

In tracing "The Development of our Knowledge of Anæsthesia," the subject of the toast, the speaker briefly alluded to the crude anæsthetic practices of the ancient, mediæval, and modern world, and commented upon the fact that although mandragora, hemp, opium, and hyoscyamus, the agents chiefly relied upon to produce anæsthetic effects in earlier times, were dangerous when given in full narcotic doses, still the stupefying effects of alcohol had been known to man from a remote antiquity, and might have been systematically employed for anæsthetic purposes as has been successfully done in our own day. Ether, too, had been known for five hundred years before its employment by Morton, and nitrous oxide gas for fully seventy years before its use by Wells.

With such agencies at command the speaker thought the long delay in securing the systematic practice of anæsthesia among the most puzzling problems presented to the student of human history, but said that while we might wonder at the slow development of anæsthesia in the past, future generations would possibly find equal cause for wonderment that we remain oblivious to truths and principles which, to them, will appear so simple and obvious.

That even now we are far from having any accurate conceptions of how or why anæsthetics anæsthetize, although, as far as the cruder mechanism of the processes is concerned, Bernard's theory of the partial coagulation of the protoplasmic constituents of nervous tissue is probably the true one.

Our knowledge concerning the progressive sequences of these effects upon the centres of intellection, co-ordination, movement, sensation, respiration, and circulation have not undergone any material modification since first described by Flourens in 1847.

As a result of the carefully conducted experiments of the Hyderabad Commission, the previously generally accepted view that chloroform is an agent having a specifically depressant power over the cardiac motor ganglia has undergone modifications, it having been conclusively shown that the fall in blood-pressure in chloroform narcosis is the result not of cardiac paralysis, but of capillary dilatation through the vaso-motor system. The speaker thought that a knowledge of this fact might tend to diminish the number of fatalities from the agent in question, as the more thoroughly the true effects of any drug are understood the more intelligently and effectively it can be employed.

Dr. Litch alluded to the discovery of the anæsthetic power of cocaine locally applied, and to the fact that even that agent was not entirely safe, as several fatalities and many cases in which dangerous symptoms have occurred are on record, and expressed the opinion that the ideal anæsthetic for prolonged operations yet remained to be discovered,—an agent perfectly safe, perfectly effective, and entirely convenient of administration.

The speaker thought that were it not for mechanical difficulties the mixture of nitrous oxide with free oxygen, as being both lethal and life-giving, would closely approximate the ideal sought for, and that a removal of these difficulties was quite among the possibilities of the future.

In conclusion, Dr. Litch called attention to the possible perversions of the use of anæsthetics and pain-obtunding agents in general, and expressed the opinion that the age is growing too timid in regard to pain and too clamorous for drugs which, at whatever cost, shall give immediate relief from even the slightest suffering.

"The Medico-Legal Aspect of Anæsthesia" was the topic given to District Attorney George S. Graham, Philadelphia.

This address was far above the average of such speeches. It abounded in wit and brilliant thought.

His description of a visit made to Dr. J. D. Thomas to have a tooth removed under anæsthesia was appreciated by the dental fraternity. "Now," he said, "I went there with the expectation that my demise was a very possible result;" and when the doctor said, "Now breathe gently, now a little harder," he thought of the before-mentioned possibility, and then suddenly he heard seraphic music and he felt so glad that, as he was surely dying, he was ascending instead of *going down*. He was brought out of this blissful contemplation by a rude shake and the doctor's call, "Wake up!" And then he found that the angel-music proceeded from a music-box.

Rev. S. D. McConnell, rector of St. Stephen's Church, Philadelphia, spoke on "The Humanitarian Aspect of Anæsthesia." He expressed his great pleasure in being invited to talk on this subject, for he knew of nothing that he would prefer more to do than to honor this great discovery that had been such a blessing to humanity.

Dr. G. Q. Colton, of New York, then spoke briefly, giving some excellent advice, ending by quoting admirably a passage from Shakespeare.

Mr. Charles Wells, of Hartford, Conn., followed in a tribute to the work of his father. He mentioned that he was but five years old at the time his father was actively engaged in the work, but he retained very vivid recollections of some of the events of the time.

It was past the hour of midnight when the company very reluctantly departed.

It was the universal opinion that this day had been one of those epoch-making periods that come seldom in the history of professions and peoples, but when they do appear they mark a dividing line between the old and the new.

The only regret seemed to be felt that the entire dental profession could not have been made partakers in the glad reunion and in this memorial celebration.

There was a general sentiment expressed that a determined effort should be made to secure a fund sufficient to erect a monument in Washington.

